# Non-motor correlates of wrist-worn wearable sensor use in Parkinson’s disease: an exploratory analysis

**DOI:** 10.1038/s41531-019-0094-4

**Published:** 2019-10-02

**Authors:** Daniel J. van Wamelen, Shweta Hota, Aleksandra Podlewska, Valentina Leta, Dhaval Trivedi, Alexandra Rizos, Miriam Parry, K. Ray Chaudhuri

**Affiliations:** 1 King’s College London, Department of neurosciences, Institute of Psychiatry, Psychology & Neuroscience, De Crespigny Park, London, SE5 8AF UK; 2 Parkinson Foundation Centre of Excellence, King’s College Hospital, Denmark Hill, London, SE5 9RS UK; 3 Donders Institute for Brain, Cognition and Behaviour, Radboud University Medical Centre, Nijmegen, The Netherlands; 4 School of Psychology, University of Kent, Canterbury, CT2 7PM Kent, UK; 5 University of Milan, L. Sacco Hospital, Milan, Italy

**Keywords:** Outcomes research, Parkinson's disease

## Abstract

Wearable sensors are becoming increasingly more available in Parkinson’s disease and are used to measure motor function. Whether non-motor symptoms (NMS) can also be measured with these wearable sensors remains unclear. We therefore performed a retrospective, exploratory, analysis of 108 patients with a diagnosis of idiopathic Parkinson’s disease enroled in the Non-motor Longitudinal International Study (UKCRN No. 10084) at King’s College Hospital, London, to determine the association between the range and nature of NMS and an accelerometer-based outcome measure of bradykinesia (BKS) and dyskinesia (DKS). NMS were assessed by the validated NMS Scale, and included, e.g., cognition, mood and sleep, and gastrointestinal, urinary and sexual problems. Multiple linear regression modelling was used to identify NMS associated with BKS and DKS. We found that BKS was associated with domains 6 (gastrointestinal tract; *p* = 0.006) and 8 (sexual function; *p* = 0.003) of the NMS scale. DKS was associated with domains 3 (mood/cognition; *p* = 0.016), 4 (perceptual problems; *p* = 0.025), 6 (gastrointestinal tract; *p* = 0.029) and 9 (miscellaneous, *p* = 0.003). In the separate domains, constipation was significantly associated with BKS. Delusions, dysphagia, hyposmia, weight change and hyperhidrosis were identified as significantly associated with DKS. None of the NMSS domains were associated with disease duration (*p* ≥ 0.08). In conclusion, measures of BKS and DKS were mainly associated with gastrointestinal problems, independent of disease duration, showing the potential for wearable devices to pick up on these symptoms. These exploratory results deserve further exploration, and more research on this topic in the form of comprehensive large-scale studies is needed.

## Introduction

Current PD management guidelines focus predominantly on treating motor symptoms and evidence for the treatment of non-motor symptoms (NMS) is lagging behind the treatment of motor symptoms.^1,2^ Addressing NMS is crucial as some can present 15–20 years before the onset of motor symptoms and impact greatly on quality of life.^3,4^ Moreover, there is a clear need for objective monitoring of NMS as many of these symptoms are unreported by up to 42.8% of NMS that were undeclared by PD patients in the clinic, which were later identified by the NMS Questionnaire.^5^

The Parkinson’s KinetiGraph™ (PKG), developed by Global Kinetics Corporation, is a wrist-worn wearable sensor device that uses an accelerometer to provide continuous ambulatory monitoring of movement.^6^ The device is worn on the primarily affected arm and produces bradykinesia (BKS) and dyskinesia (DKS) scores every 2 min, which are plotted over several days to create a graphical representation of the patient’s motor control.^6^ The PKG algorithms for BKS and DKS have been validated against the Unified Parkinson’s Disease Rating Scale (UPDRS) Part III and IV, and the Abnormal Involuntary Movement Scale (AIMS).^7^ The device has MHRA and FDA–CEA marking, and is one of the recent objective wearables measuring BKS and DKS in PD.^7,8^

Wearable sensor devices measuring motor outcomes have previously been used to identify associations with NMS in PD.^8–12^ A study using wrist-worn actigraph monitors found that circadian rest-activity rhythms were associated with cognitive performance, independent of sleep.^13^ In addition, the PKG has already been utilised to measure NMS in a clinical setting. There are data to support its efficacy to address impulse control disorder and sleep disorders in PD.^9–12^ Periods of immobility measured by the PKG were in concordance with the detection of sleep by daytime polysomnography, suggesting that ‘percentage time immobile’ could be a measure of daytime sleepiness in PD.^11^ Similarly, a conditioned response to acknowledge the consumption of medication on the PKG device has been associated with high impulsive–compulsive behaviour scores.^10^ In the current study, we aim to explore other non-motor correlates of the PKG.

## Results

### Patient characteristics

A total of 108 participants (72 male and 36 female) for whom a PKG readout was available were included in the analysis. In our centre, we provide wearable sensor monitoring to all patients who are willing to use the PKG watch for six consecutive days. Patients were not selected based on the presence of motor fluctuations, and their baseline non-motor characteristics did not differ from patients participating in the NILS study who did not receive a PKG (*p* = 0.91), making a selection bias unlikely. In total, 80 participants had a PKG recording within 30 days of their clinical assessment (74.1%), 6.5% between 31 and 60 days, 9.3% between 61 and 90 days, and 10.2% between 91 and 120 days. Participant demographics are summarised in Table 1.

**Table 1:** Participant demographics and non-motor scales and questionnaires

	Mean	SD	Range
Age (years)	62.8	9.4	38.0–82.0
Age at onset (years)	55.3	9.1	35.0–77.0
Disease duration (years)	7.5	5.5	0.0–26.0
Hoehn and Yahr stage	2.9	1.0	2–4
LEDD (mg)	950.4	673.8	0–2818
SCOPA			
Motor score	10.9	5.7	0–30
Activities of daily living	6.2	4.3	0–20
Motor complications	2.5	2.6	0–11
Parkinson’s Kinetigraph measures			
Bradykinesia score	27.0	8.7	6.9–52.9
Dyskinesia score	5.6	12.9	0.0–84.7
NMSS			
Total scores	57.2	37.8	0–161
1. Cardiovascular	1.8	3.1	0–16
2. Sleep/fatigue	12.0	8.7	0–36
3. Mood/cognition	9.2	11.3	0–48
4. Perceptual/hallucinations	2.3	5.3	0–32
5. Attention/memory	6.7	8.6	0–36
6. Gastrointestinal tract	5.0	5.9	0–24
7. Urinary	7.2	9.1	0–36
8. Sexual function	2.5	5.5	0–24
9. Miscellaneous^a^	10.4	7.6	0–36
PDSS score	98.2	25.9	34–149
HADS (anxiety) score	7.5	4.3	0–18
HADS (depression) score	6.4	3.9	0–19
MMSE score	28.2	3.6	23–30
ESS score	9.1	5.4	0–23

*SCOPA* Scales for Outcomes in Parkinson’s Disease, *LEDD* levodopa equivalent dose, *NMSS* non-motor symptom scale, *PDSS* Parkinson’s disease sleep scale, *HADS* hospital and anxiety depression scale, *MMSE* mini mental state examination, *ESS* Epworth’s sleep scale, *PKG* Parkinson’s KinetiGraph, *BKS* bradykinesia score, *DKS* dyskinesia score

^a^The miscellaneous domain contains questions about pain, hyposmia, hyperhidrosis and weight change

### Bradykinesia

A multiple linear regression model was created with the BKS score as the dependent variable and disease duration, age of onset, LEDD, NMSS domains 1–9, MMSE, HADS, PDSS and ESS scores as independent variables (Table 2). The results of the regression indicated that the predictors explained 24.8% of the variance in the model (adjusted *R*^2^ = 0.248, SE = 7.23). BKS was predicted by NMSS domain 6 (gastrointestinal tract; *β* = 0.303, *p* = 0.006), NMSS domain 8 (sexual function; *β* = 0.301, *p* = 0.003) and LEDD (*β* = −0.327, *p* = 0.001). After exclusion of patients on apomorphine and levodopa intestinal infusion therapy, this was the only NMSS domain 6 (*β* = 0.389, *p* = 0.008, other dependent variables *p* > 0.09) in patients on oral therapies (*n* = 80).

**Table 2:** Multiple linear regression model of bradykinesia and dyskinesia non-motor predictors defined by the non-motor symptom scale domains

	BKS		DKS	
	*β*	*P*	*β*	*P*
Disease duration	0.047	0.62	0.088	0.36
Age	0.072	0.45	0.036	0.71
NMSS cardiovascular	−0.080	0.44	0.065	0.54
NMSS sleep/fatigue	−0.006	0.96	0.083	0.52
NMSS mood/cognition	0.075	0.54	−0.308	**0.016**
NMSS perceptual/hallucinations	−0.055	0.58	0.229	**0.025**
NMSS attention/memory	−0.102	0.34	−0.055	0.62
NMSS gastrointestinal tract	0.303	**0.006**	−0.247	**0.029**
NMSS urinary	0.100	0.37	0.024	0.83
NMSS sexual function	0.301	**0.003**	0.005	0.96
NMSS miscellaneous^a^	−0.182	0.09	0.322	**0.003**
PDSS	0.182	0.11	0.059	0.61
HADS	−0.009	0.95	0.144	0.29
MMSE	−0.029	0.76	−0.116	0.23
ESS	0.062	0.56	−0.139	0.20
LEDD	−0.327	**0.001**	0.258	**0.009**

*NMSS* Non Motor Symptom Scale, *LEDD* levodopa equivalent dose, *PDSS* Parkinson’s disease sleep scale, *HADS* hospital and anxiety depression scale, *MMSE* mini mental state examination, *ESS* Epworth’s sleepiness scale, *BKS* bradykinesia score, *DKS* dyskinesia score

^a^The miscellaneous domain contains questions about pain, hyposmia, hyperhidrosis and weight change

Significant values are highlighted in bold

In order to control for the possible effect of NMS changes over time, we performed a separate analysis for patients who had the start of the PKG recording taking place within 30 days of their clinical assessment (*n* = 80). This model (adjusted *R*^2^ = 0.436, SE = 7.28) confirmed that BKS was predicted by NMSS domain 6 (gastrointestinal tract; *β* = 0.241, *p* = 0.042), NMSS domain 8 (sexual function; *β* = 0.304, *p* = 0.010) and LEDD (*β* = −0.398, *p* = 0.001).

To further clarify which specific NMS predicted BKS, we performed multiple linear regression with the specific items within NMSS domains 6 and 8 added as independent variables. We observed that only item 21 (constipation) was a significant predictor of BKS (*β* = 0.311, *p* = 0.001).

When the same linear regression was repeated, but with SCOPA motor scores as the dependent variable, again only gastrointestinal symptoms were identified as the only significant predictor (*p* = 0.045; model *R*^2^ = 0.30). Moreover, none of the NMSS domains showed a significant association with disease duration (*p* ≥ 0.08).

### Dyskinesia

A multiple linear regression model was created with the DKS score as the dependent variable and the aforementioned demographic and non-motor-scale predictors as independent variables (Table 2). The predictors explained 33.2% of the variance in the model (adjusted *R*^2^ = 0.332, Std. error = 10.51). DKS was predicted by NMSS domain 3 (mood/cognition; *β* = −0.308, *p* = 0.016), NMSS domain 4 (perceptual problems/hallucinations; *β* = 0.229, *p* = 0.025), NMSS domain 6 (gastrointestinal tract; *β* = −0.247, *p* = 0.029), NMSS domain 9 (miscellaneous; *β* = 0.332, *p* = 0.003) and total LEDD (*β* = 0.258, *p* = 0.016). In patients on oral therapies (*n* = 80), DKS was predicted by NMSS domain 4 (*β* = −0.262, *p* = 0.037), NMSS domain 6 (*β* = −0.406, *p* = 0.002) and NMSS domain 9 (*β* = 0.259, *p* = 0.048), but not by LEDD (*β* = 0.151, *p* = 0.19).

In order to determine which particular NMS within the aforementioned domains were significant in predicting DKS, a second multiple linear regression model was created, with the specific items in domains 4, 6 and 9 included as independent variables. In this model, NMSS items 9 (anxiety; *β* = −0.296, *p* = 0.008), 14 (delusions; *β* = 0.400, *p* = 0.001), 20 (dysphagia; *β* = −0.278, *p* = 0.005), 28 (hyposmia; *β* **=** 0.187, *p* = 0.032), 29 (weight change; *β* = 0.205, *p* = 0.027) and 30 (hyperhidrosis; *β* = 0.273, *p* = 0.004) were found to be significant predictors of DKS.

In order to control for the possible effect of NMS changes over time, we performed a separate analysis for patients who had the start of the PKG recording taking place within 30 days of their clinical assessment (*n* = 80). This model (adjusted *R*^2^ = 0.484, SE = 10.89), in line with the findings from the total cohort, showed that DKS was predicted by NMSS domain 3 (mood/cognition; *β* = −0.520, *p* < 0.001), NMSS domain 4 (perceptual problems/hallucinations; *β* = 0.282, *p* = 0.010), NMSS domain 9 (miscellaneous; *β* = 0.215, *p* = 0.045) and LEDD (*β* = 0.347, *p* = 0.002).

## Discussion

This study addressed the impact of wearable sensor monitoring on the assessment of NMS. The main finding in our exploratory analyses revealed that PKG BKS scores were associated with low LEDD, constipation and the sexual domain scores of the NMSS. In addition, DKS scores on PKG correlated with high LEDD, mood and cognition, perceptual problems, constipation and the miscellaneous symptom domain of the NMSS. These results support the concept that wearable sensor use may potentially serve as a marker for some key NMS in PD.^14,15^

The relationship between LEDD and BKS supports the concept of BKS being a key hypo-dopaminergic symptom of PD further supported by the fact that lower LEDD was associated with worse BKS and SCOPA motor scores. These findings support previous studies showing the usefulness of PKG in reflecting BKS in patients and that increasing dopaminergic medication may thus reduce BKS, a clinically known phenomenon.^6^ Clinically, these data thus support the use of PKG to optimise medication regimes in ‘undertreated’ patients as has also been recently reported.^16^ However, of key interest is the fact that we report that constipation, regarded as a NMS of gastrointestinal dysfunction^4^ and sexual dysfunction is in general associated with higher BKS scores. Large-scale studies are still required to further investigate this relationship. The clinical impact could be that high BKS scores on the PKG should prompt clinicians to enquire about and address gastrointestinal symptoms in their patients. Previous studies have shown that 43.5% of gastrointestinal symptoms are unreported by patients; possible reasons include a lack of understanding that these symptoms are related to PD, embarrassment to disclose these symptoms and clinic time being focused on the discussion of motor symptoms instead.^5^ Identifying possible non-motor surrogate markers of the PKG could benefit wider clinical practice in this way.

DKS, on the other hand, was related to high LEDD, consistent with DKS being a hyper-dopaminergic symptom and has been well described in relation to PKG use.^17,18^ Interestingly, high DKS scores were associated with euphoria and increased perceptual problems and hallucinations.^17,18^ Such an occurrence is in line with evidence suggesting that excessive dopaminergic stimulation in the mesocortical and mesolimbic dopaminergic pathways results in elevated mood and euphoria, as well as psychotic symptoms such as delusions and hallucinations.^19^

This study underlines a pivotal unmet need in PD in that accurate and objective assessment of NMS is still lacking. One can argue that the results from our study can be replicated through careful history taking for example by specifically asking about constipation in patients who are very bradykinetic. However, current assessments of NMS in PD, but also of motor symptoms, and subsequent treatment decisions continue to suffer from inaccuracies related to clinical scales as well as recall bias and retrospective data entry for diaries.^20^ Using wearable sensors may address part of this problem and surely needs further exploration, even though, in the long term, wearable sensor use may not emerge as a robust marker for the heterogeneous NMS of PD.

There are of course limitations in this work. While the PKG assessments were based on a 6-day recording, the non-motor assessments included in the regression model were only completed at one point in time. Like the motor symptoms, these NMS also fluctuate^18^ and may not provide an accurate depiction of the duration, severity and course of the symptoms. Moreover, patient-based assessments are prone to subjectivity and recall bias and clinician-based assessments are prone to inter-rater variability. In addition, not all participants in the NILS study had a PKG reading close to their assessment date. We matched the PKG reading to the closest NILS assessment, selecting an arbitrary cut-off of 4 months. The NMS, and also the LEDD, may have improved or worsened during these 4 months. When we, however, analysed the group of patients who had a PKG recording within 30 days of their clinical assessment, we did not find differences in the main outcomes, making it unlikely that the longer-time PKG and clinical assessment have influenced our results. Finally, it might be argued that our findings were driven by disease duration as a confounder. However, a separate analysis showed none of the studied NMS to be associated with disease duration.

In summary, gastrointestinal function (particularly constipation) was significantly associated with BKS scores of PKG, providing evidence that the PKG could be used to monitor non-motor outcomes, such as constipation. Although the observation that, e.g., constipation is linked to general BKS is not novel, this study shows that motor outcomes obtained through wearable sensors are associated with such NMS. This suggests that further work should be undertaken to explore the role of wearable sensors to be used as markers of specific NMS in PD.

## Methods

### Patient cohort

All consecutive patients between July 2014 and February 2018 with a diagnosis of probable idiopathic PD who participated in the Non-motor Longitudinal International Study (NILS) and had a baseline assessment were included in the study. The NILS cohort study (adopted as a national study by the National Institute of Health Research in the United Kingdom (UKCRN No. 10084)) is a comprehensive study with non-motor profiling of PD as the primary outcome measure addressing natural history of NMS, treatment response and clinico-pathological-imaging correlations. For this analysis, only patients who had an assessment at King’s College Hospital were included. The NILS study was authorised by local ethics committees (NRES South East London REC, 10084, 10/H0808/141). All patients gave written consent prior to study procedures in accordance with the Declaration of Helsinki. PKG readings were performed as part of standard clinical care after the device was granted licence in the United Kingdon from 2016. All patient data were anonymised and coded. The project was also under the data protection act code of the General Data Protection Regulation (2016/679 EU) (GDPR).

### Data collection

Data extracted from the NILS database concerned sex, disease onset (in years), Hoehn and Yahr stage, levodopa equivalent dose (LEDD), Scales for Outcomes in Parkinson’s Disease (SCOPA) motor, NMS Scale (NMSS),^21^ Hospital Anxiety and Depression Scale (HADS),^22^ Parkinson Disease Sleep Scale (PDSS),^23^ Epworth Sleepiness Scale (ESS)^24^ and Mini Mental State Examination (MMSE) scores.^25^ The NMSS has 30 items and nine domains: cardiovascular (2 items), sleep/fatigue (4 items), mood/cognition (6 items), perceptual problems/hallucinations (3 items), attention/memory (3 items), gastrointestinal tract (3 items), urinary function (3 items), sexual function (2 items) and miscellaneous (4 items). Each item scores on a multiple of severity (from 0 to 3) and frequency scores (from 1 to 4) and the theoretical range of the NMSS total score is 0–360. The NMSS domains were defined as follows: cardiovascular and falls (domain 1), sleep/fatigue (domain 2), mood/cognition (domain 3), perceptual problems/hallucinations (domain 4), attention/memory (domain 5), gastrointestinal tract (domain 6), urinary (domain 7), sexual function (domain 8) and miscellaneous (domain 9). The aforementioned scales and outcomes were performed during the ON-state in patients.

The PKG output provided the BKS and DKS data for each patient. The PKG system consists of a data logger containing a rechargeable battery, a triaxial accelerometer and memory to store data, and it is worn on the wrist of the most affected side. The PKG is worn continuously for six consecutive days, after which data are downloaded and analysed by using a proprietary algorithm to calculate the BKS and DKS scores. The BKS score is the median value of BKS scores over the period from 05:00 to 21:00 of each recorded day and BKS correlates with UPDRS III scores. The DKS score is the median value of DKS over the period from 05:00 to 21:00 of each recorded day and the score correlates with the modified Abnormal Involuntary Movement Score assessed at the time of PKG.^6,26^ Baseline values for BKS and DKS are 18.6 and 4.3, respectively.^8^ Optimal control in PD is defined as a BKS score of 23 or less and a DKS score of 7 or less, whereas poor control is defined by a BKS score of 25 or higher and a DKS score of 9 or above.^8^

**Fig. 1:**
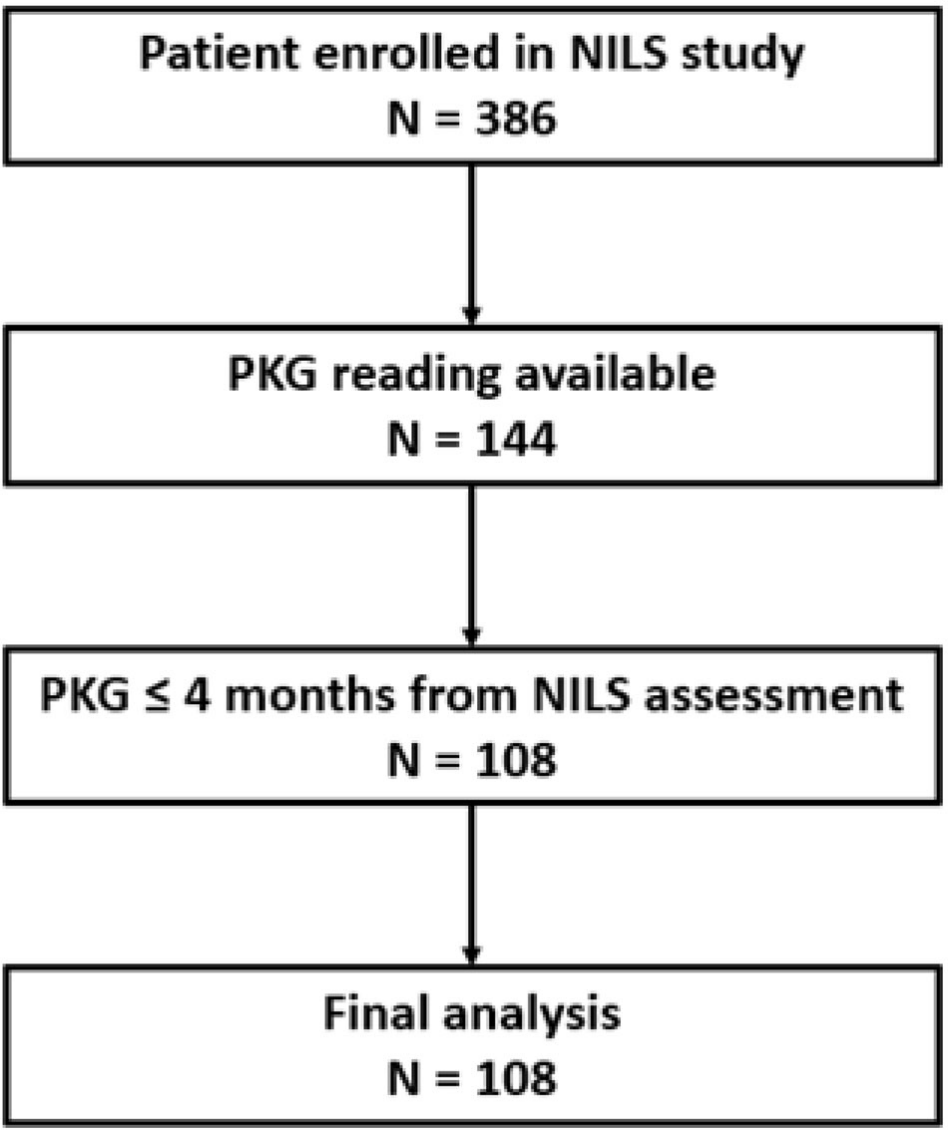
Flowchart of patient inclusion

All patients participating in the NILS study (*n* = 386) for whom a 6-day PKG reading was available between July 2014 and February 2018 were assessed for inclusion into the study (*n* = 144). A total of 108 participants were identified with a PKG reading within 4 months of their NILS assessments and fulfilling all other criteria (Fig. 1).

### Outcome measures and statistical analysis

The primary outcome in the study was to assess whether the PKG measures BKS and DKS are associated with NMS. The secondary outcome was to explore which of the NMS domains were associated with BKS and DKS outcomes. For these, we used multiple linear regression with BKS or DKS as dependent variables and with PD demographics (age at onset, disease duration and LEDD), NMSS domains 1–9, PDSS, MMSE, HADS and ESS scores as independent variables. Data are presented as mean ± standard deviation or percentage when appropriate. All data were analysed by using SPSS Version 24 (IBM SPSS Statistics for Windows, Version 24.0, Armonk, NY: IBM Corp.).

### Reporting summary

Further information on research design is available in the Nature Research Reporting Summary linked to this article.

## Supplementary information

Reporting Summary

## Data Availability

All data that support the findings of this study are available from the corresponding author upon reasonable request.

## Code availability

Data analysis were performed and coded by using SPSS Version 24 (IBM SPSS Statistics for Windows, Version 24.0, Armonk, NY: IBM Corp.). The code for running the regression analyses can be found at https://www.ibm.com/uk-en/analytics/spss-statistics-software.
